# Proteome-wide curation of experimentally validated HPV T-cell epitopes identifies key gaps in our understanding of cellular immunity to HPV and informs vaccine design

**DOI:** 10.3389/fimmu.2026.1917170

**Published:** 2026-08-28

**Authors:** Syandrez Prima Putra, Selin Cankat, Leo Swadling

**Affiliations:** 1 Institute of Infection Immunity and Transplantation, University College London, London, United Kingdom; 2 Department of Microbiology, Faculty of Medicine, Universitas Andalas, Padang, Indonesia; 3 Center for Infectious Disease Diagnostic and Research (PDRPI), Faculty of Medicine, Universitas Andalas, Padang, Indonesia

**Keywords:** conservation, cross-reactivity, human papillomavirus, T-cell epitope, vaccine design

## Abstract

**Background:**

Human papillomavirus (HPV) drives both malignant and benign tumours. Current prophylactic vaccines are type-restricted, not optimised for T-cell induction, and lack therapeutic efficacy. Although T-cells are critical for both preventing and clearing HPV infection, experimentally validated HPV T-cell epitopes remain fragmented across the literature, limiting systematic evaluation of cellular immune targets.

**Methods:**

We curated experimentally validated HPV T-cell epitopes from the Immune Epitope Database (IEDB). Epitopes were mapped across HPV proteins and genotypes, and analysed for response rate, sequence conservation across 454 representative HPV genomes, and HLA restriction patterns.

**Results:**

485 unique experimentally validated HPV epitopes have been described (133 studies; 1,494 functional assays). Consistent with research focus and viral biology, E6 and E7 proteins account for >60% of known HPV epitopes despite accounting for ~10% of the viral proteome. High-risk HPV types, especially HPV16 and HPV18, were the most studied (*p* <.001) and were enriched for CD8^+^ epitopes (*p* <.001). We identified major knowledge gaps, including: underrepresentation of structural proteins such as L2; limited epitope coverage for low-prevalence HPV genotypes; a bias towards common HLA alleles. *In silico* analysis indicated greater conservation of epitopes in L1/L2 and across high-risk HPV types. Conserved, commonly detected, and HLA-promiscuous epitopes were highlighted and we provide panels of candidate epitopes for consideration in immune monitoring, broad-spectrum prophylactic vaccines, and high-risk targeted therapeutic vaccines.

**Conclusion:**

This study provides the first comprehensive atlas of experimentally validated HPV T-cell epitopes and ranked epitope candidates for translational application. We demonstrate that our understanding of HPV T-cell immunity is constrained by biases in antigen, genotype and HLA focus and by incomplete epitope mapping. Addressing these gaps will be essential for a comprehensive assessment of cellular immunity and for utilising T-cells in next-generation vaccines.

## Introduction

Human papillomavirus (HPV) is a circular double-stranded DNA virus with an ~8 kb genome and is responsible for approximately 5% of all human cancers, including cervical, vulvar, vaginal, penile, anal, and head and neck (1). HPV-related cervical cancer remains a leading cause of cancer-related mortality amongst women globally, especially in settings with limited access to vaccination and screening (2). HPV comprises more than 450 distinct genotypes when defined by <90% sequence identity in the L1 gene relative to other known types, of which 12 genotypes (HPV16, 18, 31, 33, 35, 39, 45, 51, 52, 56, 58, and 59) are classified as carcinogenic or high-risk types by the International Agency for Research on Cancer (IARC) (3). The development and global deployment of prophylactic bivalent, quadrivalent, and nonavalent L1-based virus-like particle vaccines has the potential to prevent 70-90% of HPV-related cancers associated with vaccine-covered genotypes (4). However, these vaccines are type-restricted, non-universal, and lack therapeutic efficacy against established infection or disease. In low- and middle-income countries, where HPV-associated cancers remain highly prevalent, vaccination coverage is limited by cost and infrastructure, and screening and treatment programmes are often insufficient, resulting in a substantial residual disease burden even for vaccine-covered genotypes (5, 6). Together, these limitations highlight the need for next-generation HPV vaccines that are cross-protective, affordable, and have both prophylactic and therapeutic applications.

One promising approach lies in leveraging T-cell immunity (7–9). Beyond neutralising antibodies, T-cells play a crucial role in controlling viral infections by eliminating infected cells (10). Several lines of evidence point to a key role for T-cell immunity in controlling HPV infection and associated neoplasia (11). HPV-specific T-cells can be found in the blood and at higher numbers in the tumour site and draining lymph node in HPV infected individuals (9, 12). Those who progress to cervical intraepithelial neoplasia have a lower magnitude T-cell response than those who don’t (12, 13). Expansion of HPV-specific T-cells has also been temporally correlated with regression of warts (14). Genetic association studies have repeatedly found associations between outcome and human leukocyte antigen (HLA), in particular for class II alleles (15–17) and HIV positivity and associated defects in cellular immunity are associated with higher rates of HPV acquisition, persistence and cancer progression (18, 19).

We, and others, have recently shown that T-cells can protect against detectable viral infection even in the absence of antibodies (20–23), in particular memory T-cell responses targeting conserved viral regions, as observed in abortive SARS-CoV-2 infection (24). These observations establish a general immunological principle whereby T-cell immunity targeting conserved viral regions can in some circumstances be sufficient to mediate cross-variant and potentially infection-blocking protection (25). T-cells can recognise all viral proteins - including highly-conserved non-structural regions that are not accessible to antibodies, are inherently cross-reactive, and tend to be more long-lived than humoral immunity (8, 26, 27). This highlights the potential of T-cells to provide broad and more durable anti-viral protection. Applying this principle beyond SARS-CoV-2 provides a conceptual framework for HPV, whose viral proteome comprises early proteins (E1–E7), involved in replication and oncogenesis, and late proteins (L1, L2), forming the viral capsid (28). As with SARS-CoV-2, T-cell epitopes derived from conserved and potentially functionally constrained regions of the HPV proteome may enable cross-genotype immunity by targeting regions that are less subject to immune escape (24, 29). HLA molecules present viral peptides to T-cells, and their polymorphism determines population-level immunity as demonstrated in previous HPV studies (30). Comprehensive mapping of HPV T-cell epitopes across viral proteins, HPV types and HLA-restriction is therefore critical to identify the most promising candidates for broad-spectrum vaccine design. We hypothesised that the currently described HPV T-cell epitope repertoire reflects research bias as much as biological immunodominance, and that identifying key gaps will allow us to better understand the contribution of T-cells to HPV control and to better utilise them therapeutically.

To begin to address this gap, we curated experimentally validated HPV T-cell epitopes from published studies. The Immune Epitope Database (IEDB) provides the most comprehensive and standardised repository (primarily added by IEDB staff curators) of experimentally validated T-cell epitopes (https://www.iedb.org) (31), enabling reproducible large-scale analyses across pathogens. Our focus was threefold: to chart known epitope distribution across proteins and genotypes, to evaluate conservation against a reference panel of complete genomes, and to assess HLA-promiscuity. This approach enables an assessment of how extensively HPV T-cell epitopes have been explored to date and where key gaps remain. It also acts as a resource for individuals designing reagents (peptide pools, MHC multimers) to investigate cellular immunity to HPV in natural history or vaccine studies. Ultimately, this analysis aims to guide the prioritisation for future T-cell studies in HPV, to highlight epitopes of interest for next-generation HPV vaccines, whilst also laying the groundwork for broader application to other oncogenic viruses.

## Methods

### Study design, data collection and initial curation strategy

**Figure 1:**
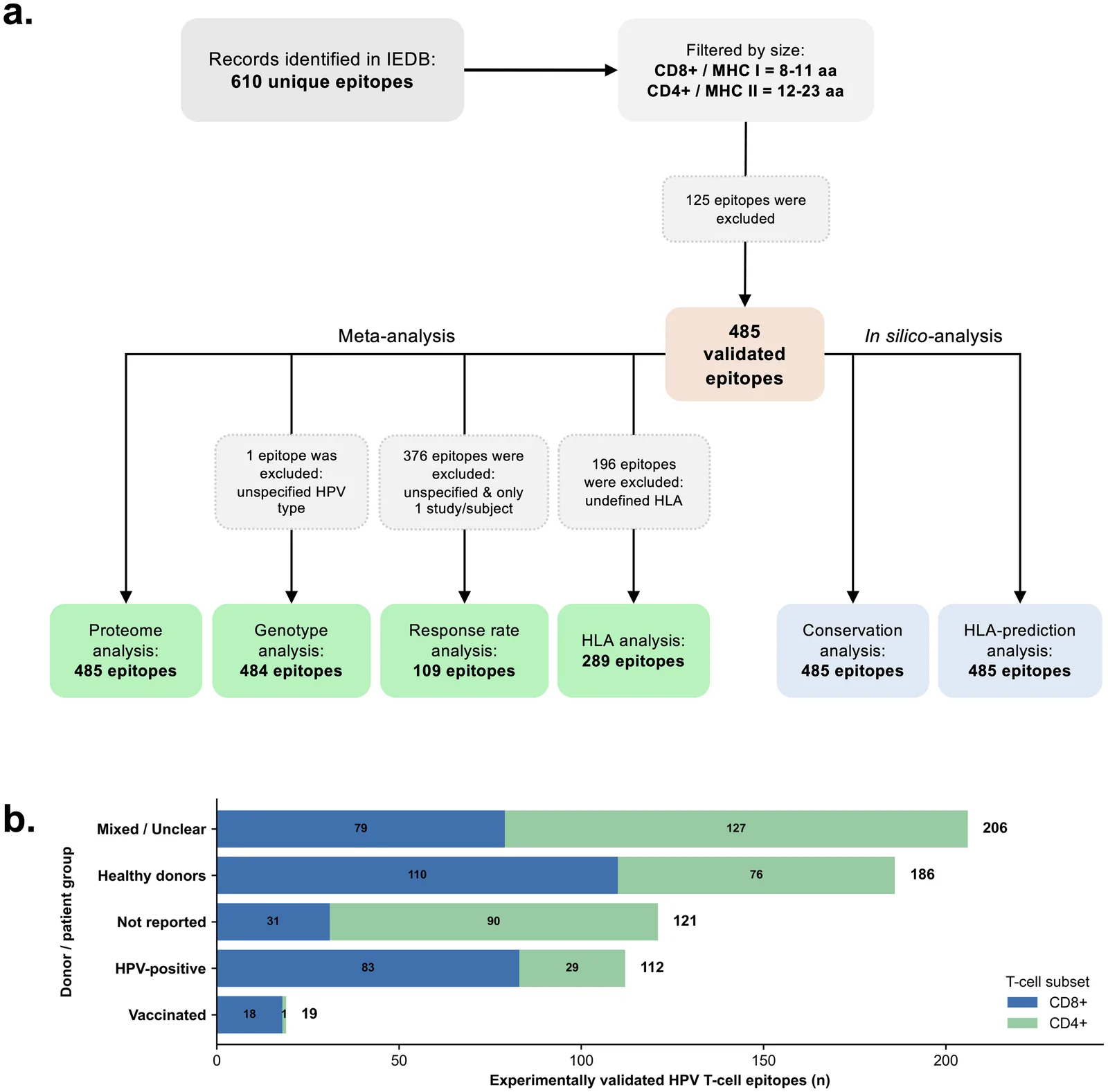
Study workflow and characteristics of experimentally validated HPV T-cell epitopes. **(A)** Overview of data curation and downstream analyses. A total of 610 unique HPV T-cell epitopes were identified from IEDB. After filtering by epitope length (CD8^+^ MHC-I, 8–11 aa; CD4^+^ MHC-II, 12–23 aa), 485 experimentally validated epitopes were retained for analysis. These epitopes were subsequently used for proteome, genotype, response-rate, HLA restriction, conservation, and HLA-prediction analyses. **(B)** Distribution of experimentally validated HPV T-cell epitopes according to donor population and T-cell subset. Bars indicate the number of unique CD4^+^ and CD8^+^ epitopes reported in vaccinated individuals, HPV-positive individuals, healthy donors, mixed/unclear cohorts, or studies in which donor status was not reported. Data were obtained from IEDB (http://www.IEDB.org) based on queries on August 14th, 2025.

To systematically characterise the currently reported and experimentally validated T-cell epitope landscape of HPV (excluding any epitopes without functional T-cell assay data), we performed a retrospective meta-analysis of epitope data curated from the Immune Epitope Database (IEDB) (https://www.iedb.org) (31), complemented by *in silico* analyses (**Figure 1A**). For the inclusion criteria, we searched the database using the following filters (1): filter options = T-cell (excluding B-cell and MHC binding-only assays) (2), epitope = any (3), epitope source, organism = Human papillomavirus (all types) (4), T-cell assay outcome = positive (5), MHC restriction = any (6), host = *Homo sapiens* (human) (7), disease = any, and (8) reference = any. We exported the results from the ‘Assays’ query as full data columns. To maintain consistency, we merged epitopes with identical peptide sequences across studies and counted each sequence once as a unique epitope for distribution analyses, whilst preserving all associated assay information and references. We then screened the peptides by length, retaining only 8–11 amino acids for Major Histocompatibility Complex (MHC) Class I restricted CD8^+^ T-cell epitopes and 12–23 amino acids for MHC Class II restricted CD4^+^ T-cell epitopes, and excluded those outside these ranges (32). We used this curated dataset as a core for analysis (485 epitopes, ‘proteome analysis’ epitope list). To minimise bias, screening and extraction were repeated three times.

We downloaded the HPV16 reference sequence FASTA file from NCBI GenBank (NC_001526.4) and extracted the protein annotations to construct a representative HPV proteome map, showing the relative positions and amino acid lengths of each protein. Using the IEDB core dataset, we then mapped all unique epitopes onto this proteome. Protein annotations were curated directly from the core dataset. Epitope density was calculated for each HPV protein (E6, E7, E1, E2, E4, E5, L2, and L1) as the number of epitopes per 100 amino acids.

### Genotype analysis

From the ‘proteome analysis’ dataset, we further filtered unique T-cell epitopes based on HPV genotype annotation and excluded epitopes with unspecified type from genotype analysis to give the ‘genotype analysis’ epitope list (n=484). HPV risk groups were classified according to the IARC, comprising 12 high-risk types (HPV16, 18, 31, 33, 35, 39, 45, 51, 52, 56, 58, and 59), with the remaining types designated as low risk. Epitopes with a single genotype annotation were classified as single-type, whereas those associated with more than one genotype were classified as cross-type.

### Response rate analysis

From the ‘proteome analysis’ dataset, we generated a separate ‘response rate analysis’ epitope list by retaining epitopes evaluated in more than one independent study and more than one subject and with available positive-response and total-tested counts, yielding 109 epitopes. Response rate was operationally defined as the aggregate proportion of positive epitope tests reported across eligible IEDB records. For each unique epitope, counts reported across studies were summed, and response rate was calculated as the total number of positive responses divided by the total number tested (range 0–1). This measure represents the frequency of reported positive epitope tests rather than the proportion of unique individuals responding and was therefore interpreted as a descriptive measure of epitope recognition, not as a measure of within-host immunodominance. Aggregate positive-response proportions were compared between CD4^+^ and CD8^+^ T-cell epitopes and across HPV proteins. The proportional contribution of each HPV protein to the overall T-cell epitope pool was illustrated using stacked horizontal bar plots, showing the relative percentage and number (n) of epitopes per protein. Epitope response profiles were visualised using immunome browser–style plots (IEDB), where response rates were mapped against amino acid positions within each protein sequence. The numbers of studies and tested subjects are provided for each epitope, allowing users to apply more stringent evidence filters where required.

### HLA analysis

Epitopes from the ‘proteome analysis’ dataset were further filtered based on HLA (MHC class) annotation. All epitopes without defined HLA were excluded from the analysis, leaving 289 epitopes in the ‘HLA analysis’ epitope list. Epitope subsets were stratified into known MHC class I (CD8^+^) and MHC class II (CD4^+^) annotations according to IEDB data. The numbers of epitopes with known HLA-annotated and allele-level restrictions were presented in absolute counts. To characterise HLA promiscuity, epitopes annotated with a single HLA allele were classified as mono-allelic, whereas those with more than one allele were classified as multi-allelic or HLA-promiscuous epitopes. Candidate epitopes for vaccine development were prioritised if they demonstrated a response rate >0.5 and were restricted by more than one HLA allele.

### Conservation analysis

We sought to identify whether currently known T-cell targets cluster in highly variable or highly conserved regions of the HPV proteome. To complement the experimental epitope dataset, each unique T-cell epitope identified from the proteome analysis (n=485) was mapped *in silico* to 454 distinct HPV genomes with known GenBank identifiers retrieved from the Papillomavirus Episteme (PaVE; https://pave.niaid.nih.gov/explore/reference_genomes/human_genomes; **Supplementary Table 1**) (33). The original experimentally validated IEDB epitope annotations were retained throughout the analysis, whilst the *in silico* mapping was used solely to extend these observations by predicting epitope distribution and conservation across the broader PaVE genome collection. This analysis was used to estimate HPV type coverage and sequence conservation across all known HPV diversity and to provide additional information on conservation breadth beyond the original IEDB-derived dataset.

To assess epitope conservation across the current known diversity of HPV sequences, we included all genomes curated by PaVE. This dataset comprised 155 reference genomes recognised by the HPV Reference Center, 71 genomes pending approval, and 228 additional genomes that have not been formally classified by the HPV Reference Center but that exhibit <90% L1 sequence identity to all recognised HPV types and have therefore been included in PaVE. We intentionally included both reference and non-reference PaVE genomes to capture the broadest currently available HPV sequence diversity. This approach enabled the identification of regions that have remained conserved despite HPV diversification, potentially highlighting functionally constrained targets for T-cell immunity. The resulting datasets can also be sub-setted to include only high-risk HPV types when conservation within clinically relevant genotypes is of primary interest.

Mapping was performed using six-frame translation to capture epitopes encoded on both forward and reverse strands. Conservation was defined as a 100% amino acid match. For downstream analyses, conservation was summarised at the HPV type level: epitope conservation was expressed as absolute counts (N) and percentages (%) of HPV types in which the epitope sequence was found identically across all 454 reference genomes. Conservation was further stratified by peptide length, protein, and T-cell type (CD4^+^ or CD8^+^). Statistical comparisons of conservation distributions were performed for proteins, T-cell subsets and risk groups. The top 40 HPV types with the highest number of mapped epitopes were identified, with annotation of their risk classification (high-risk vs low-risk). A proteome map was constructed to visualise the distribution of fully conserved (100%) epitopes across HPV proteins. The map was stratified by HPV risk group (high-risk vs low-risk) and further annotated according to T-cell subset (CD8^+^ or CD4^+^) and protein functional category (oncoprotein, replication, or structural).

### HLA prediction analysis

To evaluate HLA promiscuity, each unique epitope from the ‘proteome analysis’ dataset (n=485) was assessed for predicted HLA binding *in silico* using NetMHCpan (v4.1) (34) and NetMHCIIpan (v4.1) (35) following the default recommended thresholds. HLA supertypes group alleles with overlapping peptide-binding specificities, enabling population-level inference of T-cell epitope coverage using a limited set of representative molecules (36). Binding affinity to MHC class I alleles (CD8^+^) was predicted across a panel of 20 representative HLA class I molecules, selected to represent the major HLA class I supertypes: HLA-C*02:02, HLA-A*02:01, HLA-C*04:01, HLA-A*03:01, HLA-A*01:01, HLA-A*11:01, HLA-C*06:02, HLA-B*08:01, HLA-A*26:01, HLA-C*03:03, HLA-C*03:04, HLA-C*01:02, HLA-B*15:01, HLA-A*24:02, HLA-B*58:01, HLA-B*39:01, HLA-B*07:02, HLA-B*40:01, HLA-A*33:03, and HLA-B*27:05 (37). These alleles were selected to balance broad population-level immunogenetic coverage with computational tractability. For MHC class II epitopes (CD4^+^), binding was predicted across the IEDB reference panel of HLA class II molecules (including HLA-DRB1*07:01, HLA-DQA1*01:02/DQB1*06:04, HLA-DQA1*02:01/DQB1*02:01, HLA-DRB1*09:01, HLA-DPA1*01:03/DPB1*02:01, HLA-DQA1*01:02/DQB1*05:01, HLA-DPA1*01:03/DPB1*04:01, HLA-DRB1*13:01, HLA-DRB1*01:01, HLA-DQA1*01:03/DQB1*05:01, HLA-DRB1*03:01, HLA-DPA1*01:03/DPB1*04:02, HLA-DRB1*11:01, HLA-DRB1*15:01, HLA-DRB1*04:01, HLA-DQA1*04:01/DQB1*03:01, HLA-DQA1*05:01/DQB1*03:01, HLA-DQA1*05:05/DQB1*03:01, HLA-DRB1*04:03, and HLA-DQA1*01:02/DQB1*06:02) (37). The selected HLA allele panel was predicted to provide near-global theoretical coverage, with estimated population coverage of 97.22% for HLA class I, 99.74% for HLA class II, and 99.99% when both classes were considered together. Predicted binding affinity was expressed as percentile rank scores, with lower values indicating stronger binding. For MHC class I, epitopes were classified as strong binders (SB) when EL rank ≤0.5%, weak binders (WB) when 0.5–2%, and non-binders when >2%. For MHC class II, epitopes were classified as SB when rank ≤1%, WB when 1–5%, and non-binders when >5%. HLA binding predictions were used to assess relative HLA promiscuity and coverage rather than to infer functional immunogenicity. The ≤5% MHC class II threshold was retained for the primary analysis as a conservative threshold for candidate prioritisation. Because broader percentile-rank thresholds are also used in exploratory MHC class II epitope discovery, we additionally performed sensitivity analyses using ≤10% and ≤20% as alternative binder thresholds, whilst retaining the ≤1% strong-binder threshold (38). For each epitope, the number of predicted HLA binders (SB or WB) was calculated to quantify promiscuity. Epitope distributions were differentiated systematically for CD8^+^/MHC I and CD4^+^/MHC II subsets by: (1) non-binders versus binders, (2) mono- versus multi-allelic, (3) comparison of total HLA binders per epitope, (4) HPV proteins, and (5) the number of WB and SB in each HLA allele. Heatmaps were generated to depict the binding profiles of the top 20 promiscuous peptides within each HPV protein across the representative HLA allele panel.

### Statistical analysis and reproducibility

To ensure rigorous evaluation of the data, we applied statistical methods tailored to the type and distribution of each variable. Categorical outcomes were analysed using Pearson’s chi-square test or Fisher’s exact test, and the strength of association was expressed as odds ratios (OR) with 95% confidence intervals (CI). For continuous variables that were not normally distributed, comparisons were performed with the Mann–Whitney U test or Kruskal–Wallis test. Correlations between variables were assessed using Spearman’s rank correlation (ρ). A p-value <0.05 was considered statistically significant. For response rates, 95% CIs were estimated using the Wilson score method. All analyses were carried out in Python v3.12.11, employing established scientific libraries including pandas (v2.2.2), NumPy (v2.0.2), SciPy (v1.16.1), statsmodels (v0.14.5), matplotlib (v3.10.0), seaborn (v0.13.2), and Biopython (v1.85). Figures were produced in vector format to ensure publication quality.

## Results

### Defining a core dataset of HPV T-cell epitopes

To synthesise the known antigenic landscape of HPV we collated 610 peptide sequences reported across 133 human studies/1,494 different functional assays from the IEDB (**Figure 1A**). To ensure consistency in size, we excluded 125 peptides that did not meet peptide length criteria, resulting in a core dataset of 485 unique epitopes, consisting of 293 CD4^+^ and 192 CD8^+^ epitopes (**Supplementary Table 2**). This curated dataset highlights both the diversity of epitope sources and the availability of high-quality, functionally validated sequences. Building on this curated dataset, we next explored six key aspects of HPV epitope biology: (1) proteome distribution, (2) genotype distribution, (3) response rate, (4) HLA restriction, followed by *in silico* prediction of (5) epitope conservation and (6) HLA binding. For genotype analysis, we retained 484 epitopes after excluding one with an unspecified HPV type. For response-rate analysis, we retained 109 epitopes evaluated in more than one independent study and more than one subject and with available positive-response and total-tested counts. For HLA analysis, we focused on 289 epitopes with clearly defined HLA. Finally, *in silico* analysis was performed on all 485 epitopes. Together, these refinements provide a robust foundation for dissecting epitope conservation, breadth of recognition, and their implications for T-cell–mediated protection.

The curated dataset comprises epitopes derived from diverse study contexts, including healthy donors, HPV-positive patients, mixed or unclear patients, vaccination studies, and incompletely annotated cohorts (**Figure 1B**), reflecting the heterogeneous nature of the available HPV T-cell epitope literature. Epitopes described only in vaccine studies may, in some cases, reflect peptides that are not processed and presented during natural infection; however, these were a small subset of the known epitopes described here (19/485).

### Proteome-wide mapping highlights understudied regions and high epitope density of E6/E7

**Figure 2:**
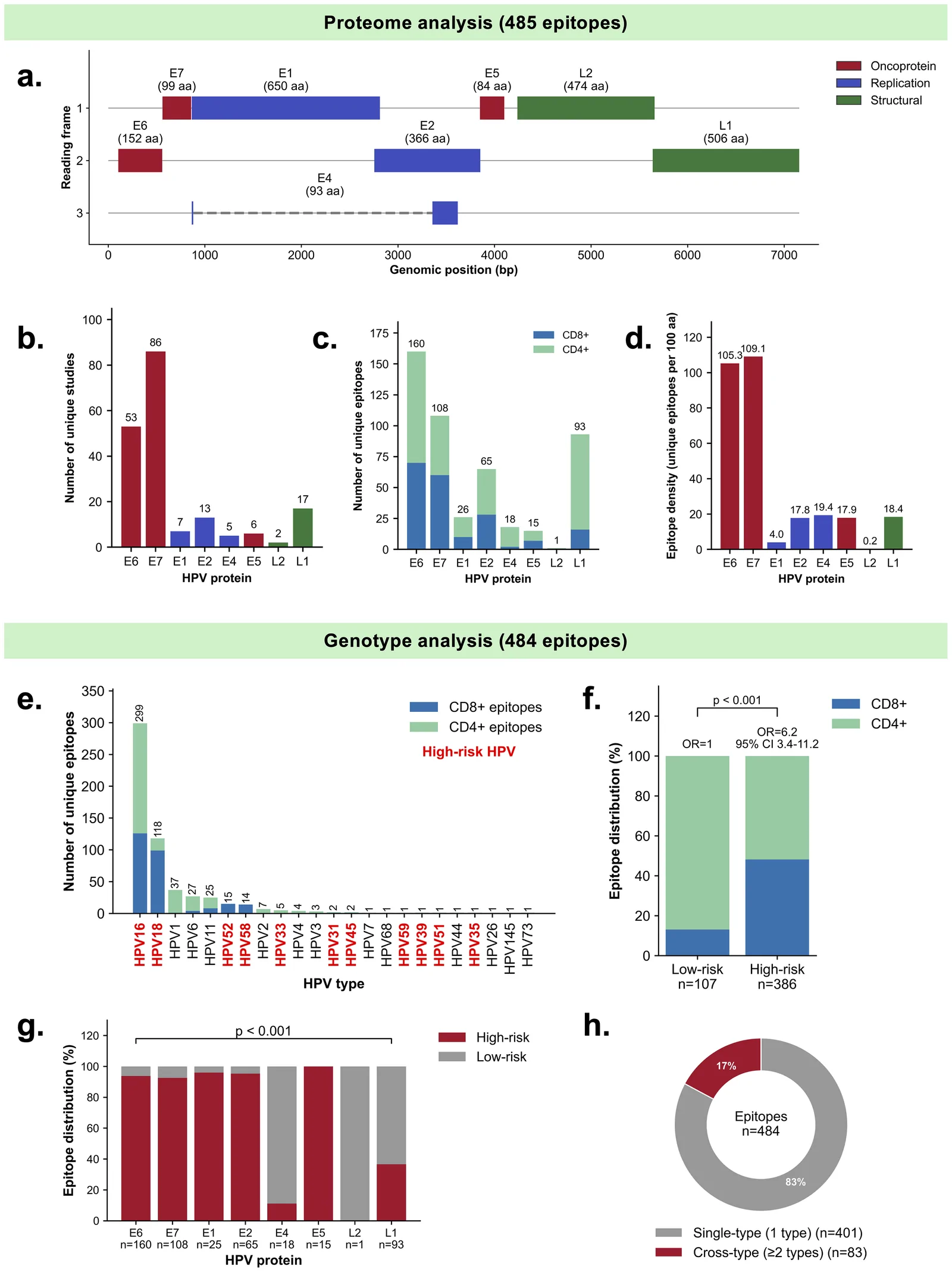
Proteome-wide and genotype distribution of experimentally validated HPV T-cell epitopes. Proteome and genotype analyses were performed using 485 and 484 experimentally validated HPV T-cell epitopes, respectively, curated from the Immune Epitope Database (IEDB). **(A)** Schematic overview of the HPV16 reference genome and proteome showing the relative genomic positions and approximate protein lengths of major HPV proteins grouped into oncogenic, replication-associated, and structural proteins. **(B)** Number of unique studies reporting experimentally validated epitopes for each HPV protein. **(C)** Distribution of validated CD8^+^ and CD4^+^ epitopes across HPV proteins. **(D)** Density of experimentally validated epitopes normalised to protein length (epitopes per 100 amino acids) across HPV proteins. **(E)** Distribution of validated CD8^+^ and CD4^+^ epitopes across HPV genotypes. **(F)** Relative epitope distribution between low-risk and high-risk HPV groups. Odds ratios (OR) and 95% confidence intervals are shown. **(G)** Proportional distribution of epitopes derived from high-risk and low-risk HPV types across HPV proteins. **(H)** Distribution of single-type and cross-type epitopes amongst validated HPV T-cell epitopes.

First, we explored the antigen distribution patterns of the 485 known unique functionally validated T-cell epitopes across the entire HPV proteome (**Figures 2A–D**). The proteome of HPV is composed of oncoproteins (E6, E7, E5), replication proteins (E1, E2, E4), and structural proteins (L1, L2) (**Figure 2A**). E8^E2-derived epitopes were not included in this analysis due to the absence of experimentally validated T-cell epitope data for this protein. CD8^+^ epitopes were dominated by 9-mers, whilst CD4^+^ epitopes were largely 15-mers (**Supplementary Figure 1A**). The number of studies mapping epitopes in each viral protein differed remarkably: E6/E7 were the most assayed proteins, with L2 being represented in just two studies (**Figure 2B**). E6 contributed the most epitopes for both CD8^+^ and CD4^+^ T-cells (70 and 90, respectively), followed by E7 (60 and 48) and L1 (16 and 77). In contrast, only a single epitope has been described in L2 (**Figure 2C**), despite L2 being identified as a promising candidate antigen for vaccines due to its conserved regions (39). Many CD4^+^ T-cell epitopes have been identified in the main target of currently licensed vaccines, L1, likely due to an interest in CD4^+^ help for these antibody-based vaccines.

To account for the difference in length of viral proteins, epitope density was calculated as the number of epitopes per 100 amino acids. The density of known epitopes varied widely by protein (**Figure 2D**) and no significant association was observed between protein length and epitope density across the HPV proteome (ρ=−0.52, *p* = 0.183) (**Supplementary Figure 1B**). Instead, T-cell epitope representation was dominated by the small oncoproteins E6 and E7, which emerged as clear outliers, with disproportionately high epitope density, whilst other similarly sized proteins, such as E4 and E5, showed substantially lower density of validated epitopes. To contextualise this pattern, we applied the same epitope density analysis to SARS-CoV-2 proteins, which have been extensively and systematically mapped (40). Epitope density for SARS-CoV-2 proteins fell between ~100 and 300 epitopes per 100aa over a wide range of protein lengths, without pronounced outliers like E6/E7. Proteins L2 and E1 have a lower number of known epitopes than would be expected for their size (**Supplementary Figure 1B**), and they are understudied (**Figure 2B**), highlighting them as candidate proteins for future epitope mapping. Together, these findings indicate that current HPV epitope datasets are strongly shaped by research focus on E6/E7, but that there is also likely an additional contribution from underlying viral biology, such as high and prolonged protein expression for these oncoproteins.

### High-risk HPV genotypes dominate the published T-cell epitope repertoire

We next explored how reported T-cell epitopes are distributed across genotypes and clinical risk categories. 484 unique HPV epitopes had known genotype information (**Figures 2E–H**). Most epitopes were identified in studies of high-risk HPV types HPV16 and HPV18. Significantly more epitopes were identified in studies of high-risk than low-risk types (**Figure 2E**). Known epitopes selected from high-risk types were approximately six times as likely to be CD8^+^ as CD4^+^ (OR = 6.18; 95% CI 3.40–11.22; *p* <.001) (**Figure 2F**). When stratified by protein, high-risk types were represented with increased epitope representation in E5, E2, E1, E6, and E7 but reduced epitope representation in L1, L2, and E4, compared to low-risk (*p* <.001) (**Figure 2G**). This bias is expected as there is much interest in the therapeutic potential of cytotoxic CD8^+^s targeting the oncoproteins of the high-risk genotypes. Whilst most epitopes were reported for a single genotype, approximately 17% were found to be present amongst multiple types of HPV (**Figure 2H**). Very few studies assessed the conservation of epitopes or the cross-reactivity of T-cell responses across genotypes and so this is a significant underestimation of the potential for pan-genotype epitopes. Below we compile a reference sequence dataset and perform *in silico* analysis to better assess epitope conservation. These data overall reflect a high scientific emphasis upon high-risk types of HPV, particularly for CD8^+^-restricted epitopes, but also cross-type epitopes as promising candidates for broad-spectrum vaccines.

### Oncoproteins harbour the most frequently recognised HPV T-cell epitopes

To examine reported patterns of epitope recognition across HPV proteins, we analysed aggregate positive-response proportions rather than epitope density. We included 109 epitopes evaluated in more than one study and more than one subject. Owing to variation in assays and study populations, these proportions were interpreted descriptively. As mentioned above, bias in the proteins and epitopes studied will impact this, but where epitopes have been considered in multiple studies and several donors, this can give some indication of the relative frequency of targeting for a given epitope. The number of studies and tested subjects for each epitope is provided in **Supplementary Table 2**.

**Figure 3:**
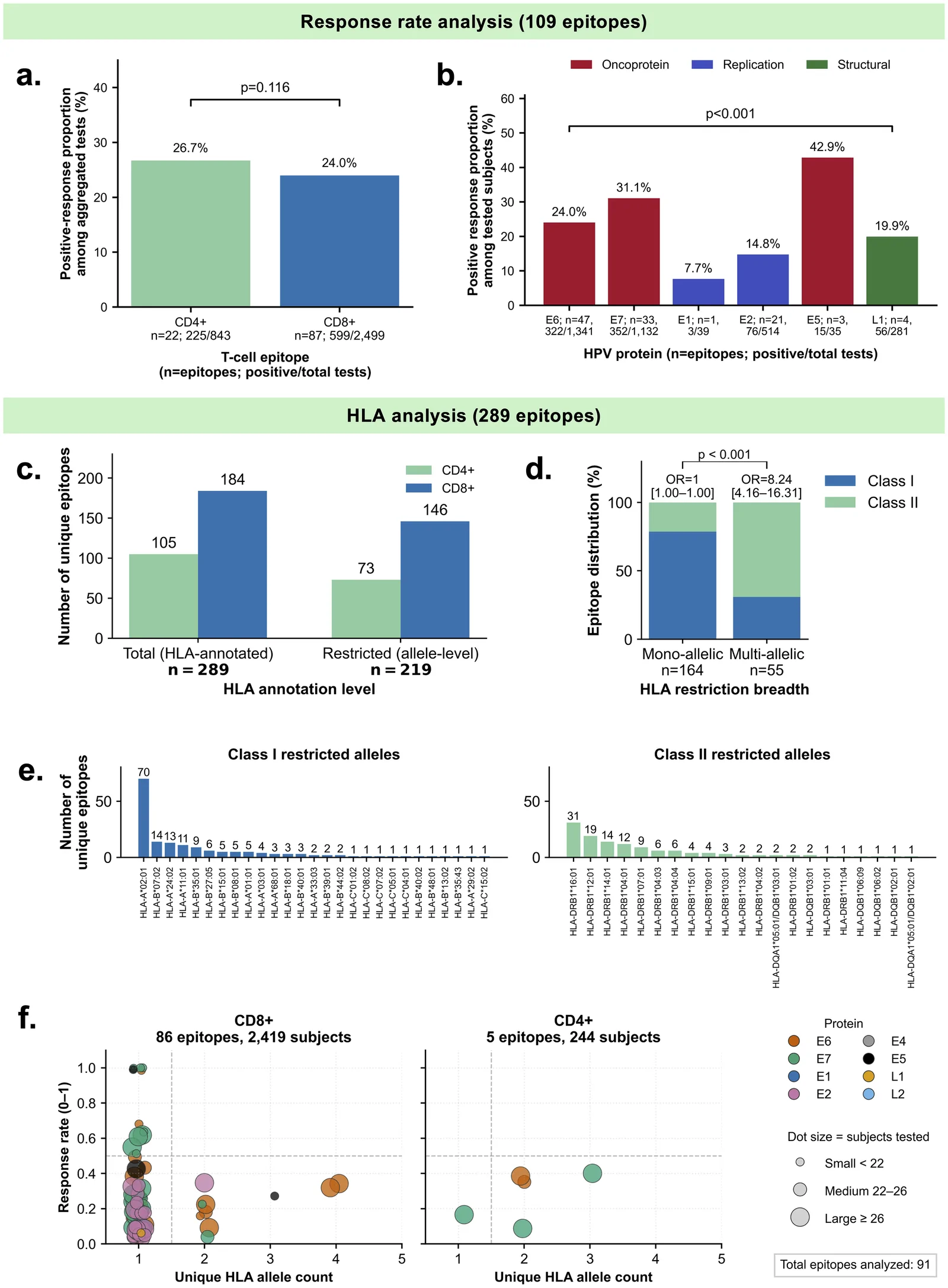
Response rate and HLA restriction landscape of experimentally validated HPV T-cell epitopes. Response rate analyses were performed using 109 epitopes with available response-rate data in multiple studies and donors, whilst HLA analyses were performed using 289 epitopes with annotated HLA information. **(A)** Aggregate positive-response proportions for validated CD4^+^ and CD8^+^ HPV T-cell epitopes. Positive/total test counts are shown below the bars. **(B)** Aggregate positive-response proportions stratified by HPV protein. Positive/total test counts are shown below the bars. **(C)** Total number of validated epitopes with annotated HLA information and epitopes with defined allele-level restriction. **(D)** Distribution of mono-allelic and multi-allelic HLA restriction breadth amongst annotated epitopes. Odds ratios (OR) and 95% confidence intervals are shown. **(E)** Distribution of experimentally validated class I and class II-restricted HLA alleles. Bars represent the number of unique epitopes associated with each HLA allele. **(F)** Relationship between experimentally observed response rates and HLA restriction breadth for validated HPV T-cell epitopes. Each bubble represents one experimentally validated epitope. The x-axis indicates the number of annotated HLA alleles associated with each epitope, whilst the y-axis represents experimental response rate. Bubble size corresponds to the number of tested subjects, and colours indicate HPV proteins. CD8^+^ and CD4^+^ epitopes are shown separately.

We compared positive-response proportions between CD4^+^ and CD8^+^ epitopes and across HPV proteins before mapping them onto the viral proteome (**Figures 3A, B**; **Supplementary Figures 1C, D**). The proportion of positive tests was 26.7% for CD4^+^ epitopes (225 positive responses amongst 843 tests) and 24.0% for CD8^+^ epitopes (599 positive responses amongst 2,499 tests) (**Figure 3A**). There was no evidence of a difference in the odds of a reported positive response between CD8^+^ and CD4^+^ epitope tests, with CD4^+^ used as the reference category (OR = 0.87, 95% CI = 0.72–1.03; *p* = 0.116). The proportion of positive tests differed across HPV proteins (p<0.001), with higher proportions observed for E5, E6, and E7 than for E1, E2, and L1 (**Figure 3B**). To investigate whether high response rate epitopes clustered in particular regions, response rates were plotted across the viral proteins (**Supplementary Figure 1C**). For several proteins (E1, E5 and L1), the limited number of reported epitopes prevented meaningful evaluation of response-rate patterns. By comparison, E6, E7 and E2 were represented by relatively better experimental evidence, allowing areas of higher epitope density to be visualised (**Supplementary Figure 1D**).

### Uneven HLA representation highlights gaps in HPV epitope discovery

To assess the potential breadth of T-cell cross-reactivity in genetically heterogeneous populations, we compared known HLA restriction profiles for 289 epitopes of HPV with available allelic data (**Figures 3C–F**; **Supplementary Figure 2**; **Supplementary Table 3**). Of the HLA-annotated epitopes, a substantial proportion could be assigned to specific allele-level restrictions, with CD8^+^ epitopes showing a higher degree of precise HLA characterisation than CD4^+^ epitopes (**Figure 3C**). However, CD4^+^ epitopes were eight times more likely to be restricted by multiple alleles (promiscuous) than CD8^+^ epitopes (OR = 8.24; 95% CI = 4.16-16.31; *p* < 0.001) (**Figure 3D**). Overall, HLA restriction profiles were unevenly distributed, with a small number of alleles accounting for most epitopes described for MHC class I and MHC class II (**Figure 3E**). A total of 27 Class I and 21 Class II HLA alleles restricted known HPV epitopes, with HLA-A*02:01 (70 epitopes) and HLA-DRB1*16:01 (31 epitopes) being the most common restricting alleles; this likely represents research bias towards alleles that are frequent in well studied populations and identifies understudied alleles. The number of epitopes identified per HLA allele did not differ significantly between Class I and Class II molecules (*p* = 0.536) (**Supplementary Figure 2A**). The number of HLAs restricting epitopes within a protein was significantly correlated with the number of epitopes described for that protein (**Supplementary Figure 2B**).

We next explored the relationship between HLA allele restriction and response rates (**Figure 3F**). The most broadly restricted CD8^+^ epitope binds 4 different HLA alleles, compared to a maximum of 3 for CD4^+^ epitopes. No epitope simultaneously met all prioritisation criteria for experimental response rate, HLA promiscuity, and supporting evidence. Whilst some epitopes demonstrated broad predicted HLA coverage and others showed relatively high response rates, these features rarely coincided within the same epitope. This limited overlap highlights the complexity of identifying broadly applicable candidate epitopes from the current HPV epitope database, calling for comprehensive mapping efforts.

### *In silico* mapping revealed limited conservation of known epitopes across HPV genotypes

**Figure 4:**
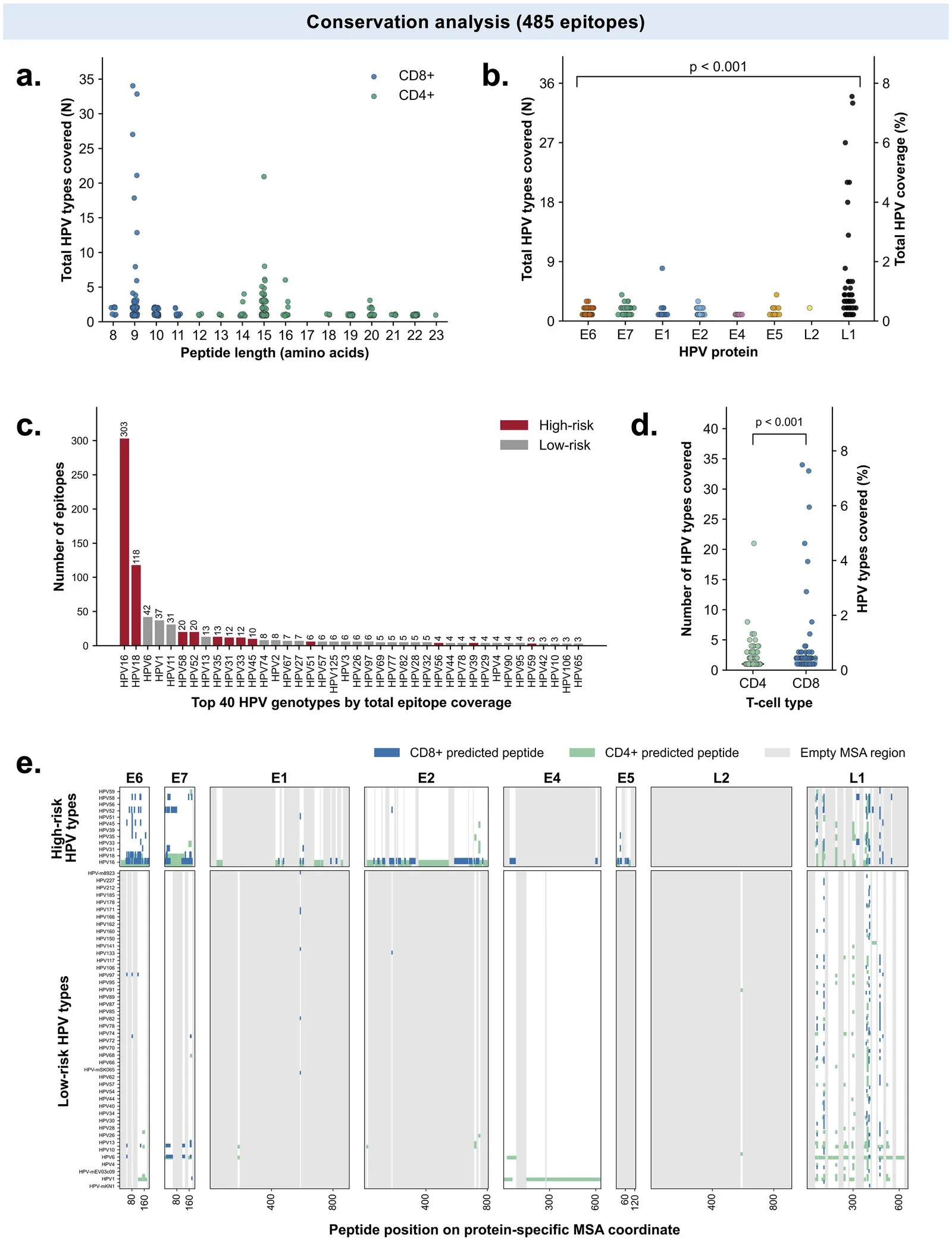
Conservation landscape of experimentally validated HPV T-cell epitopes. Conservation analyses were performed using 485 experimentally validated HPV T-cell epitopes mapped across HPV reference proteomes and multiple-sequence-alignment (MSA) coordinates. **(A)** Relationship between peptide length and total HPV types covered by exact-match analysis. **(B)** Distribution of exact-match HPV type coverage across HPV proteins. Right y-axis indicates proportional HPV coverage relative to the total analysed HPV types. **(C)** Distribution of total exact-match epitope coverage across HPV genotypes. **(D)** Comparison of HPV type coverage between CD4^+^ and CD8^+^ epitopes. Right y-axis indicates proportional HPV coverage relative to the total analysed HPV types. **(E)** Proteome-wide positional landscape of experimentally validated and predicted HPV T-cell epitopes mapped onto protein-specific MSA coordinates. Panels are separated by HPV risk group and HPV protein. Coloured regions indicate MSA-aligned positions associated with predicted CD8^+^ or CD4^+^ epitopes, whereas grey regions indicate alignment regions without mapped epitopes. This positional analysis enabled direct comparison of epitope distribution across homologous regions of different HPV types.

It is important to understand how widely HPV epitopes are shared across genotypes to facilitate the identification of candidates with broad-spectrum T-cell immunity potential. To investigate this, we retained the original HPV type annotations from IEDB and complemented them with an *in silico* mapping analysis of 485 functionally validated HPV T-cell epitopes against a database of 454 HPV genomes curated by PaVE, enabling prediction of epitope distribution across a broader range of HPV genotypes (see Methods; **Figure 4**; **Supplementary Table S4**). The maximum HPV type coverage observed was 34 genotypes (7.5%) for the CD8^+^ epitope LQFIFQLCK (9-mer) and 21 genotypes (4.6%) for the CD4^+^ epitope FNKPYWLQRAQGHNN (15-mer) (**Figure 4A**). Structural protein L1 exhibited higher conservation scores (median [IQR], 0.44% [0.22–0.66%]; p<0.001) than non-structural proteins (**Figure 4B**). No significant difference was observed between overall high-risk and low-risk epitope coverage (**Supplementary Figure 2C**). At the genotype level, HPV16 showed the greatest epitope representation, with 303 of 485 unique epitopes (62.5%) matching the HPV16 genome. Corresponding values were lower for HPV18 (118/485, 24.3%), HPV6 (42/485, 8.7%), and HPV1 (37/485, 7.6%) (**Figure 4C**). CD8^+^ epitopes exhibited significantly broader HPV type coverage than CD4^+^ epitopes (p<0.001) (**Figure 4D**). Epitope matches were unevenly distributed across the HPV proteome, with prominent clusters observed within E6 and E7 of high-risk HPV types and within the structural protein L1, whereas replication proteins contained comparatively fewer mapped epitopes (**Figure 4E**). High-risk HPV types also exhibited a greater concentration of shared CD8^+^ and CD4^+^ epitopes than low-risk HPV types, highlighting regions with potential relevance for broad vaccine design. Response rates did not differ significantly between predicted binders and non-binders for CD8^+^ epitopes (*p* = 0.272) or CD4^+^ epitopes (p=0.438) (**Supplementary Figure 2D**). Overall, most experimentally reported HPV epitopes displayed limited cross-genotype exact-match coverage.

### Supertype-based HLA prediction identifies candidate broadly presented HPV epitopes

**Figure 5:**
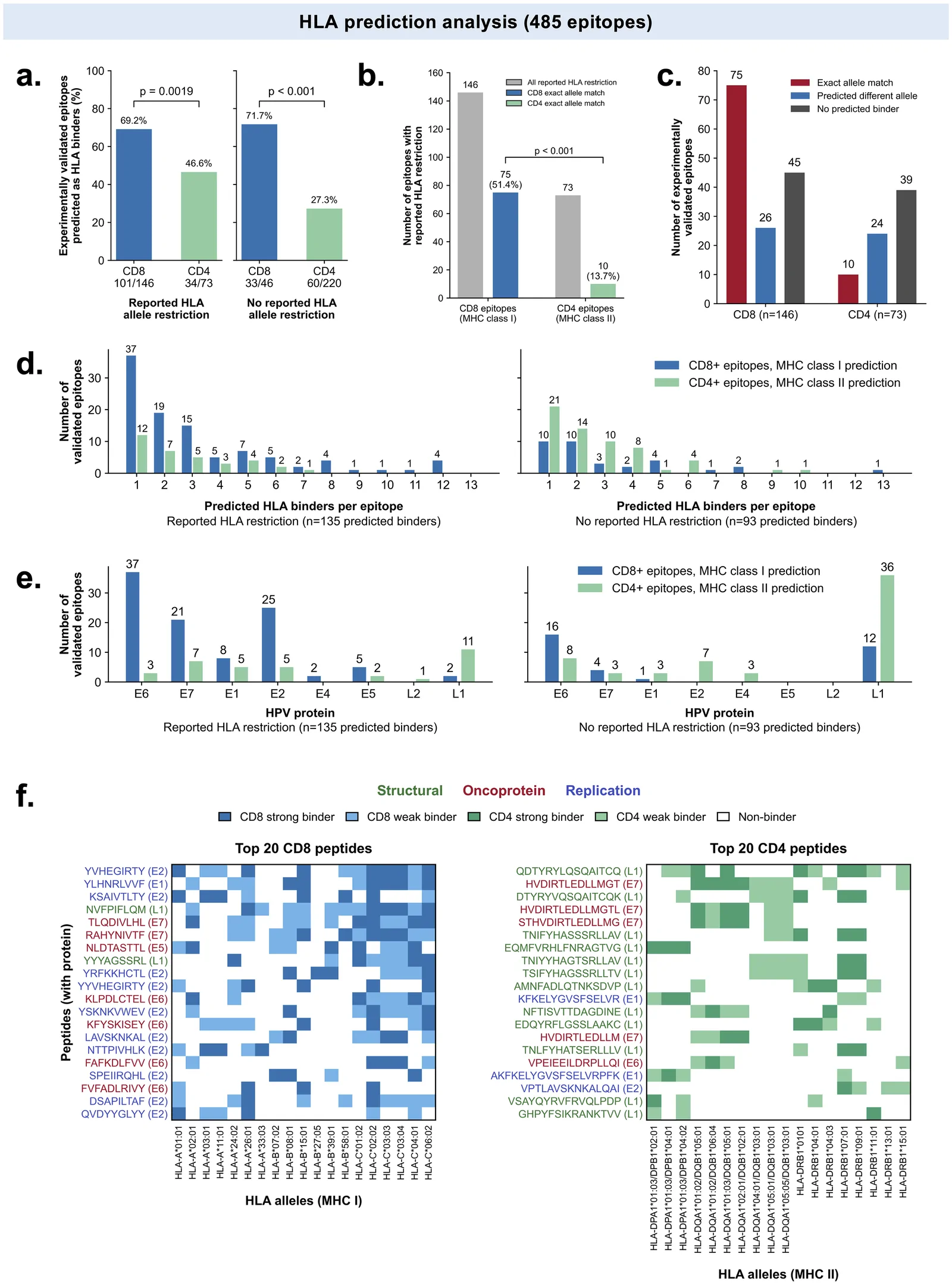
Predicted HLA-binding landscape of experimentally validated HPV T-cell epitopes. In silico HLA-binding analyses were performed for 485 experimentally validated HPV T-cell epitopes using NetMHCpan and NetMHCIIpan. CD8^+^ epitopes were analysed using MHC class I prediction thresholds, whereas CD4^+^ epitopes were analysed using MHC class II prediction thresholds. **(A)** Proportion of CD8^+^ and CD4^+^ epitopes predicted to bind at least one HLA allele, shown separately for epitopes with and without experimentally reported HLA restriction. **(B)** Number of epitopes with reported HLA restriction eligible for allele-level concordance analysis and the corresponding numbers showing an exact match between the experimentally reported and predicted HLA allele. Percentages indicate the proportion of exact allele matches within each T-cell subset. **(C)** Concordance between experimentally reported HLA restriction and in silico prediction results. Epitopes were classified as exact allele match, predicted binding to a different allele, or no predicted binder. **(D)** Distribution of predicted HLA-binding breadth amongst CD8^+^ and CD4^+^ epitopes, shown separately for epitopes with and without experimentally reported HLA restriction. The x-axis indicates the number of predicted HLA binders per epitope, and the y-axis indicates the number of epitopes. **(E)** Distribution of predicted HLA-binding epitopes across HPV proteins, shown separately for epitopes with and without experimentally reported HLA restriction. Bars indicate the number of predicted binders derived from each HPV protein. **(F)** Predicted HLA-binding profiles of the top-ranked CD8^+^ and CD4^+^ epitopes. Heatmaps show predicted weak and strong binding interactions across representative HLA class I and class II alleles. Epitope-label colours indicate structural, oncogenic, and replication-associated HPV proteins.

To systematically assess HLA-binding breadth beyond single-allele annotations which are common in IEDB, we predicted binding of known HPV epitopes to a curated panel of alleles, selected as globally common alleles that represent the major HLA supertypes (groupings of HLA alleles based on repertoire of presented peptides), using NetMHCpan and NetMHCIIpan (**Figure 5**; **Supplementary Figure 3**; **Supplementary Table 5**). Of 219 epitopes with reported allele-level HLA restriction, at least one complete reported HLA restriction was represented in the prediction panel for 133 of 146 CD8^+^ epitopes and 38 of 73 CD4^+^ epitopes (**Supplementary Figures 3A, B**). Across the full dataset, CD8^+^ epitopes were more frequently predicted to bind at least one allele within the panel compared to CD4^+^ epitopes, both amongst reported (*p* = 0.0019) and non-reported HLA subsets (*p* <.001) (**Figure 5A**). Across all epitopes with reported allele-level HLA restriction, exact concordance between reported restriction and predicted binding was significantly higher for CD8^+^ than CD4^+^ epitopes (p<.001) (**Figures 5B, C**), with allele-level analysis indicating that some mismatches reflected alternative predicted binders within related supertypes (**Supplementary Figures 3C–F**).

Predicted HLA promiscuity was next analysed separately for epitopes with reported (n=219) and without reported HLA allele restriction (n=266) (**Figures 5D, E**). CD8^+^ epitopes more frequently demonstrated multi-allelic binding, with some peptides predicted to bind up to 12 alleles in the reported HLA subset and up to 13 alleles in the non-reported subset, whereas CD4^+^ epitopes were generally restricted to fewer alleles (**Figure 5D**). Protein-level stratification showed that predicted CD8^+^ epitopes were most frequently observed in E6 and E7 across both subsets. In contrast, CD4^+^ epitopes were more evenly distributed across proteins in the reported HLA subset but showed relative enrichment in E6 and L1 in the non-reported HLA subset (**Figure 5E**). We visualised the top 20 most promiscuous CD8^+^ and CD4^+^ epitopes using heatmaps to illustrate predicted multi-allelic binding patterns across the supertype panel (**Figure 5F**). These potentially highly promiscuous peptides were derived from multiple HPV proteins, although their distribution remained uneven across the proteome. At the allele level, HLA-C*02:02 and HLA-A*02:01 accounted for the highest number of predicted class I binders, whilst HLA-DRB1*07:01 and HLA-DQA1*02:01/DQB1*02:01 accounted for the highest number of class II binders (**Supplementary Figures 3E, F**). Together, these findings indicate that the supertype framework can be used to nominate potentially broadly presented HPV T-cell epitopes, particularly for MHC class I. However, the low exact allele-level concordance, especially for MHC class II, limits predictive certainty and highlights the need for experimental validation of individual peptide–HLA interactions.

### Distinct T-cell vaccine objectives across the HPV disease continuum

The experimentally validated HPV T-cell epitope landscape revealed distinct patterns of antigen representation, response rate, and conservation across the viral proteome. E6 and E7 accounted for the majority of reported epitopes and remained dominant following analyses of HLA restriction and response frequency, whereas several epitopes derived from L1, L2, E1, and E2 demonstrated broader conservation across HPV genotypes. These study-derived patterns of antigen representation, response frequency, HPV genotype conservation, and predicted HLA-binding breadth, considered alongside established evidence regarding stage-specific HPV protein expression and T-cell-mediated control, informed a results-driven framework for distinct HPV vaccine objectives (**Table 1**). Conserved proteins were considered relevant for broad-spectrum prophylactic strategies because of their broader genotype representation, whereas early proteins may be relevant for targeting infected cells before malignant transformation. In contrast, E6 and E7 remained the dominant antigens within the experimentally validated epitope landscape and therefore represent key targets for therapeutic cancer vaccine approaches.

**Table 1:** Results-informed antigen and epitope considerations for distinct HPV T-cell vaccine objectives.

Clinical objective	Desired immune mechanism	Preferred antigen targets	Key epitope properties	Evidence basis
Prophylactic infection-blocking	Rapid recall of CD4^+^ and CD8^+^ memory T-cells in acute infection, preventing chronicity	All conserved proteins	Pan-HPV conservation, immunogenicity in early phase of life cycle and HLA allele breadth for global coverage	Refs. 10, 21, 22, 25, 29, 41, 42
Clearance of persistent infection	Cytotoxic clearance of infected epithelium	Early proteins	Pan-HPV conservation, immunogenicity during chronic infection and HLA allele breadth	Refs 7, 11, 14, 43–45
Therapeutic cancer vaccines	Tumour-associated antigen-driven CD8^+^ cytotoxicity ± CD4^+^ help	E6 & E7 proteins	High expression levels and presentation of epitopes in transformed cells, conservation in high-risk types and HLA allele breadth	Refs 9, 43–50

Proposed immune mechanisms are literature-informed, whereas antigen and epitope priorities are linked to the protein representation, response rate, conservation, and predicted HLA-binding analyses presented in **Figures 2**-**5**. HLA-binding predictions indicate potential presentation breadth and should not be interpreted as evidence of functional immunogenicity.

### Panels of experimentally validated HPV T-cell epitopes for immune monitoring or incorporation in vaccine design

To prioritise experimentally validated HPV T-cell epitopes for different translational applications, we integrated curated IEDB data with *in silico* analyses of HPV genotype conservation and predicted HLA binding. Three candidate panels were generated using distinct prioritisation frameworks reflecting their intended applications: (1) a broad-spectrum prophylactic panel, in which epitopes were first filtered to include only those covering more than one HPV genotype and predicted to bind at least two HLA alleles, then shortlisted hierarchically according to the number of HPV genotypes covered, the number of high-risk HPV genotypes represented, and predicted HLA-binding breadth; (2) a therapeutic panel, in which only E6- and E7-derived epitopes covering at least one high-risk HPV genotype and predicted to bind at least two HLA alleles were retained and subsequently shortlisted according to high-risk HPV coverage, predicted HLA-binding breadth, and overall HPV genotype coverage; and (3) an immune-monitoring panel, in which epitopes were required to have been evaluated in more than one independent study, tested in more than one subject, exhibit a positive response rate, and be predicted to bind at least one HLA allele, after which they were shortlisted hierarchically according to the number of independent studies, the number of tested subjects, reported response rate, and predicted HLA-binding breadth. Applying predefined biological selection criteria identified 74 epitopes for a prophylactic broad-spectrum panel, 56 for a therapeutic high-risk panel, and 73 for an immune-monitoring panel, yielding a total of 203 candidate epitopes (**Supplementary Table 6**). All panels would benefit hugely from a concerted effort to map epitopes across proteins, genotypes and HLA alleles underrepresented in the current data as highlighted above.

To assess the effect of broader MHC class II binding thresholds on epitope prioritisation, the analysis was repeated using percentile-rank cut-offs of ≤10% and ≤20%. Amongst the 293 CD4^+^ epitopes, the number predicted to bind at least one HLA class II allele increased from 94 at the primary ≤5% threshold to 142 and 189 at ≤10% and ≤20%, respectively. The number predicted to bind at least two alleles increased from 61 to 97 and 156, respectively. The corresponding panel sizes were 74, 80, and 87 epitopes for the prophylactic panel; 56, 67, and 90 for the therapeutic panel; and 73, 75, and 76 for the immune-monitoring panel (**Supplementary Table 7**).

## Discussion

HPV is a major health priority, causing anogenital and oropharyngeal cancers globally despite effective prophylactic vaccines. Current vaccines, though remarkably effective against vaccine-included types, offer limited cross-type coverage and no therapeutic efficacy (54). There is increasing evidence that T-cell immunity can act early to abort viral infections before they are established (21, 22, 25) as well as mediating viral clearance during established infections, making T-cell epitopes central targets for next-generation prophylactic and therapeutic vaccines. Prophylactic approaches should benefit by moving beyond a narrow focus on neutralising antibodies, to additionally exploiting the durability and cross-reactive potential of T-cell immunity. There are also many settings in which accurate quantification and assessment of the T-cell response to HPV may aid in our understanding of pathogenesis and immune correlates of protection. In this context, our analyses provide a framework for prioritising HPV regions and types that remain experimentally unexplored, and we highlight panels of known epitopes which can already be incorporated into vaccine design.

This study offers the first proteome-wide database of known HPV T-cell epitopes that has been curated and thoroughly annotated by protein, genotype, and HLA restriction, extending prior reports of HPV epitopes (55–58). Comparable efforts in alternate viral systems, i.e., influenza virus and SARS-CoV-2 (59, 60), have demonstrated the value of systematic epitope compilation and underscore the importance of such an enterprise for HPV. Given the heterogeneous study contexts represented in the IEDB, including healthy donors, HPV-infected patients, and vaccinated individuals, the observed epitope landscape reflects a combination of underlying viral biology and prior experimental focus, rather than a comprehensive measure of intrinsic immunogenicity across the HPV proteome. This interplay itself is, however, informative, revealing substantial gaps in epitope mapping across the HPV proteome, particularly for conserved structural and replication-associated proteins (e.g., L1 and E1). It can also highlight epitopes with a ‘relatively’ higher response rate where similar numbers of studies and individuals are considered.

HPV oncogenes E6 and E7 were highly represented amongst the epitope datasets, with peak epitope density reported despite their small size. This enrichment is consistent with long-standing evidence that E6 and E7 are frequent T-cell targets, uniformly preserved throughout tumour cells in expression profiles (46–48, 61, 62). Interestingly, when compared to comprehensively mapped SARS-CoV-2 proteins E6 and E7 remained outliers, with considerably higher number of epitopes than would be expected for their size. Notably, independent immunopeptidomics and expression-level studies have similarly reported predominant MHC presentation of peptides derived from E6/E7, with only occasional detection of other early proteins such as E1 (9), supporting a biological basis for their prominence beyond historical research prioritisation alone. Epitopes within E6/E7 were skewed towards CD8^+^ T-cell responses and high-risk HPV genotypes, a pattern that has informed the development of some prospective therapeutic vaccine candidates targeting these proteins (49–51). In contrast, proteins such as L2, have been investigated in fewer studies, translating to fewer known epitopes, despite accumulating evidence supporting their cross-type protective potential (41, 63, 64). Our previous work, which revealed minimal variation in HPV16 L2 sequences from Indonesian cervical cancer samples, supported the notion that L2 contains conserved regions worthy of further study (39). We also highlighted enrichment of CD4^+^ epitopes within L1, consistent with the extensive study of this protein in the context of licensed prophylactic antibody-based vaccines.

Consistent with its established ranking amongst the most common genotypes associated with cancers (28, 65), more epitopes have been validated for HPV16; however, it was unexpected how little was known about HPV types other than the two most prominent high-risk types, HPV16 and HPV18. The importance of identifying and exploiting CD8^+^ epitopes in oncogenes of high-risk genotypes is supported by previous work showing diminished CD8^+^ function is accompanied by impaired viral clearance and higher risk for cancers (43, 44, 66). Of particular interest is the identification of epitopes that span multiple types of HPV to signify their importance as cross-protective antigens for a vaccine, which has already been implemented amongst several viruses, such as influenza (29) and SARS-CoV-2 (67). Here we quantified conservation of known epitopes across the known HPV diversity for the first time, allowing the identification of the most conserved epitopes.

Our *in silico* analyses demonstrate that only a small percentage of known HPV T-cell epitopes exhibit broad conservation across types, with the majority being highly genotype-specific. In accordance with evidence that capsid-derived regions contain conserved epitopes that can induce cross-protection across diverse HPV types, structural proteins like L1 and L2 emerged as relatively richer sources of cross-type candidates (41, 42). Although conserved fragments within E6/E7 have been reported and are being investigated as multigenotype therapeutic targets, epitopes from early proteins tended to be type-restricted (45, 52).

Importantly, analysis of HLA restriction revealed that HPV epitopes had broad coverage of a large repertoire of Class I and Class II molecules; however, they were concentrated disproportionately within a restricted subset of alleles, most notably HLA-A*02:01 and HLA-DR4. Such distribution mirrors experimental focus on more common alleles as has been described for other viruses (37, 68). This highlights that existing HPV immunology knowledge may poorly represent large parts of the global population. The closed groove of MHC class I enforces stricter binding specificity, whilst the open-ended groove of MHC class II permits longer and more variable peptides (69). This is reflected in a preponderance towards mono-allelic restriction amongst CD8^+^ epitopes suggests narrower coverage of individual CD8^+^ epitopes than CD4^+^ epitopes, with a concern for population coverage within highly disparate genetic backgrounds (37).

Parallel HLA binding prediction showed that whilst most epitopes were predicted to bind a limited number of alleles, whereas a small subset demonstrated broad multi-allelic binding. These epitopes are especially useful because HLA promiscuity extends the range of individuals who can present and target the epitope with T-cell immunity (69, 70). The MHC class II sensitivity analysis demonstrated that predicted binding breadth was dependent on the selected percentile-rank cut-off. Broader thresholds increased the number of predicted binders and expanded the candidate panels, particularly the therapeutic panel, whereas the immune-monitoring panel was comparatively stable. We therefore retained the ≤5% cut-off for conservative primary prioritisation, as our main goal is to identify the most promising candidates for wet lab validation, and present the ≤10% and ≤20% analyses as sensitivity and exploratory assessments. The low exact allele-level concordance between experimentally reported HLA restriction and *in silico* prediction, particularly for MHC class II, indicates that predicted promiscuity should be interpreted as a hypothesis-generating measure of potential presentation breadth rather than confirmation of peptide–HLA binding. Experimental validation remains necessary before these predictions can support functional or clinical prioritisation. These findings underscore the dual challenge of limited conservation and restricted allele coverage for most HPV epitopes. Although no pan-genotype epitopes were identified here, we have highlighted a small number of epitopes with high response rates and wide HLA restriction (promiscuous) for further investigation.

Informatic approaches have been applied to the design of pan-HPV vaccines but have not yet progressed to clinical testing (45, 53, 71). Most informatic approaches use AI to predict peptide-HLA binding and/or immunogenicity of HPV peptides, without functional validation, and do not always consider conservation or HLA promiscuity and the impact these have on cross-protection and global coverage. An important emerging approach is the use of epitope prediction tools integrated with sequence conservation, but importantly with wet lab validation of epitopes, before immunogenicity and efficacy testing. As an emerging approach best practices for combining epitopes into T-cell or bivalent vaccines are to be determined; however, exciting new technologies can induce high magnitude T-cell responses (72–74), allow the testing of T-cell-inducing components alongside conventional antibody-inducing components in vaccines (e.g., adenoviruses coated in surface proteins to induce nAbs but encoding T-cell antigens (75), or viral vectors of mRNA molecules encoding Ab and T-cell epitopes (76)).

There are various limitations to this study. First, although one aim of this analysis was to identify knowledge gaps in epitope mapping, these inherent biases limit our ability to accurately determine immunodominance at the protein level and to fully characterise HLA promiscuity and cross-genotype conservation. Through highlighting these gaps, we hope to inform future efforts. By focusing on functionally validated epitopes, in particular those confirmed in multiple studies, we hope to limit the inclusion of epitopes that are not naturally processed or presented during HPV infection and tumour progression. Second, although informative, *in silico* predictions of HLA binding and conservation cannot replace experimental confirmation of T-cell responses. We cannot infer protective efficacy or the ability of polyclonal T-cell responses to cross-react and cross-protect using in silico tools; however, future mapping efforts would allow better immune monitoring and investigation of which T-cell specificities correlate with viral control, allowing rational design of vaccines to incorporate cross-protective T-cell epitopes. Third, we only considered an epitope to be pan-genotypic if sequence identity was 100%, however, T-cells have flexibility in recognition and cross-reactivity can be retained to epitope variants. Cross-reactivity and potential for cross-protection are therefore underestimated when using this strict cut-off and functional cross-recognition of variants should be performed *in vitro*. Fourth, the analyses were limited to 454 genomes. These sequences have been curated by PaVE to include International HPV reference center recognised viral types and additional putative types with <90% sequence identity to these types, in order to reflect the known papillomavirus diversity. Using single references for each type may not accurately represent intra-type variation and the true diversity of HPV. Despite the controlled curation of IEDB and further filtering on epitopes that have been observed using functional T-cell assays, there is heterogeneity in the way epitopes have been identified and a reliance on reported annotations. This heterogeneity also affects the aggregate positive-response analysis because the contributing records differed in assay type, participant population, and clinical context, whilst the absence of individual-level identifiers meant that repeated contributions by the same participants could not be excluded. Fifth, the evidence that is currently available may limit generalisability across the entire HPV spectrum by overemphasising high-risk genotypes, especially HPV16. Lastly, we are limited by our understanding of viral protein expression timing and levels. Vaccine design needs to be informed by a clear expected mechanism-of-action: prophylaxis (prevent infection), clearance (post-infection elimination of the virus/virally infected cells), therapeutic cancer treatment (post-neoplasia/cancer progression), as such vaccine-induced T-cell responses targeting a given epitope can have different efficacy according to the setting in which the vaccine is applied (7). We have highlighted candidate panels of epitopes for different settings, which can be edited as mapping is extended and our understanding of HPV biology progresses. These drawbacks illustrate how crucial it is to combine methodical *in silico* techniques with more extensive experimental research to improve epitope selection and direct the creation of epitope and bivalent vaccines that are applicable to both populations and other types.

## Conclusion

In conclusion, this study provides the first proteome-wide map of currently known HPV T-cell epitopes, integrating information on distribution, conservation, HLA restriction, and response rate. Our findings reveal the focus on E6 and E7, the underrepresentation of structural proteins such as L2, and the limited conservation and HLA coverage that constrain most known epitopes. We highlight gaps in our current knowledge of T-cell immunity and derive panels of promising candidates from known epitopes for immune monitoring or incorporation into vaccines. For rational design of epitope vaccines or bivalent vaccines for HPV, targeting both T and B-cell immunity, comprehensive mapping and stratification of T-cell epitopes by conservation and cross-reactivity are required.

## Data Availability

The original contributions presented in the study are included in the article/**Supplementary Material**. Further inquiries can be directed to the corresponding author.

## Supplementary material

Supplementary Figure 1 Additional proteome and response rate characteristics of experimentally validated HPV T-cell epitopes. Proteome analyses were performed using 485 experimentally validated HPV T-cell epitopes, whilst response rate analyses included 109 epitopes with available response-rate information in multiple studies and donors. **(A)** Length distribution of experimentally validated CD8^+^ and CD4^+^ epitopes. **(B)** Relationship between protein length and epitope density across HPV proteins, compared with previously reported SARS-CoV-2 T-cell epitope distributions. **(C)** Relative contribution of each HPV protein to the response-rate analysis dataset. **(D)** Positional response-rate profiles of experimentally validated epitopes across HPV proteins. Curves represent experimentally observed response rates mapped onto amino acid positions within each protein, illustrating variation in reported positive-response proportions across the HPV proteome.

Supplementary Figure 2 Additional HLA restriction and conservation analyses of experimentally validated HPV T-cell epitopes. HLA analyses were performed using 289 experimentally validated epitopes with annotated HLA information, whilst conservation analyses included all 485 validated epitopes. **(A)** Comparison of HLA restriction breadth between class I- and class II-restricted epitopes. Each point represents one experimentally validated epitope, and the y-axis indicates the number of reported HLA alleles associated with each epitope. **(B)** Relationship between the number of experimentally validated epitopes and the number of unique associated HLA alleles across HPV proteins. Separate analyses are shown for CD8^+^ and CD4^+^ epitopes. **(C)** Comparison of exact-match HPV type coverage between epitopes derived from high-risk and low-risk HPV types. Each point represents one experimentally validated epitope. Right y-axis indicates proportional HPV coverage relative to the analysed HPV types. **(D)** Comparison of experimental response rates between predicted HLA binders and non-binders for CD8^+^ and CD4^+^ epitopes.

Supplementary Figure 3 Additional in silico HLA-prediction analyses of experimentally validated HPV T-cell epitopes. Prediction analyses were performed for 485 experimentally validated HPV T-cell epitopes using NetMHCpan and NetMHCIIpan. CD8^+^ epitopes were analysed using MHC class I prediction thresholds, whereas CD4^+^ epitopes were analysed using MHC class II prediction thresholds. **(A)** Distribution of experimentally validated epitopes according to reported HLA restriction status and inclusion within the HLA prediction panel. **(B)** Number of experimentally validated epitopes predicted to bind at least one HLA allele within the selected prediction panels. Bars indicate epitopes with predicted HLA binding amongst panel-included epitopes for CD8^+^ and CD4^+^ datasets. **(C)** Distribution of predicted HLA binders amongst epitopes with and without experimentally reported HLA restriction. **(D)** Distribution of mono-allelic and multi-allelic predicted HLA binders across HPV proteins for CD8^+^ and CD4^+^ epitopes. **(E, F)** Distribution of predicted HLA class I and class II binders across representative HLA class I and II alleles, respectively. Bars indicate the number of experimentally validated epitopes predicted as weak or strong binders for each allele.

Supplementary Table 1 454 HPV genomes with accession numbers retrieved from PaVE database.

Supplementary Table 2 Meta-analysis of experimentally validated HPV T-cell epitopes with response rate, HLA restriction, and assay information.

Supplementary Table 3 List of experimentally validated HPV T-cell epitopes restricted by specific HLA.

Supplementary Table 4 *In silico* analysis of conservation and HLA-binding profiles of HPV T-cell epitopes.

Supplementary Table 5 *In silico* analysis of HPV epitope binders across HLA alleles.

Supplementary Table 6 Prioritised panels of experimentally validated HPV T-cell epitopes for prophylactic vaccine design, therapeutic vaccine design, and immune monitoring.

Supplementary Table 7 Sensitivity analysis of alternative MHC class II percentile-rank cut-offs.
